# Plasma albumin and calcium concentrations, and long-term mortality in primary health care patients in Sweden

**DOI:** 10.1080/02813432.2020.1843809

**Published:** 2020-11-23

**Authors:** Sofia Dalemo, Kristina Bengtsson Boström, Per Hjerpe

**Affiliations:** a Public Health and Community Medicine, Institute of Medicine, Sahlgrenska Academy, University of Gothenburg, Gothenburg, Sweden; b Regionhälsan R&D Centre, Skaraborg Primary Care, Skövde, Sweden

**Keywords:** Age groups, albumin, hypoalbuminemia, hypocalcemia, mortality, primary healthcare

## Abstract

**Objective:**

Low plasma (p)-albumin and p-calcium concentrations are associated with increased mortality in hospitalised patients. There are few studies addressing this in primary care. Low p-calcium has been associated with mortality, but it is not known whether this applies to p-albumin. Could p-albumin and p-calcium be used as markers of an increased risk of mortality?

**Purpose:**

To study p-albumin and p-calcium at baseline and their association with mortality after 10–14 years.

**Design:**

Prospective cohort study using data from a large primary health care area and the National Swedish Cause of Death Register.

**Setting:**

Primary health care in Skaraborg, Sweden.

**Subjects:**

43,052 patients (39.1% men), ≥18 years, 60.7 ± 18.4 years with p-albumin and p-calcium concentrations registered in 2001–2005.

**Main outcome measures:**

P-albumin and p-calcium concentrations at baseline and their association with mortality after a mean follow-up period of 10.3 ± 4.0 years.

**Results:**

Low p-albumin was associated with total mortality compared with normal p-albumin, greatest at lower ages (18–47 years). The hazard ratios for women and men were 3.12 (95% CI 1.27–7.70) and 4.09 (95% CI 1.50–11.14), respectively. The increased mortality was seen in both cardiovascular and malignant diseases in both women and men. In contrast, low p-calcium was not associated with increased mortality, 1.00 (95% CI 0.96–1.05). Elevated p-calcium was associated with increased mortality, 1.17 (95% CI 1.13–1.22).

**Conclusions:**

Low p-albumin could be a marker of an increased risk of mortality, especially in patients of younger ages. This finding should prompt diagnostic measures in order to identify underlying causes.

**KEY POINTS**
- Low p-albumin and calcium concentrations have been associated with increased mortality in hospitalised patients, but this is unexplored in primary care patients.
- A low p-albumin concentration at baseline was a risk marker for mortality; highest in the younger age groups.
- Increased mortality in both cardiovascular and malignant diseases was seen in both men and women with low compared with normal p-albumin concentrations.
- Elevated but not low p-calcium concentrations were associated with increased mortality after 10–14 years of follow-up.

## Introduction

Plasma (p)-albumin is an acute-phase protein synthesised in the liver. A response to serious conditions, such as cancer or cardiovascular disease, leads to decreased synthesis and transcapillary escape of albumin [1,2]. Low p-albumin concentrations can also be caused by liver damage, malabsorption or abnormal losses [3], and are associated with increased morbidity [4]. Elevated p-albumin concentrations are usually due to dehydration [3]. Men have higher but decreasing p-albumin concentrations than females up to 50 years of age. After that, the concentrations in both sexes are similar and decline with age [5]. Albumin participates in maintaining the colloid osmotic pressure and in the transportation of non-water-soluble substances, such as steroid hormones and fatty acids, but also calcium [3]. The total p-calcium concentration is thus dependent on the p-albumin concentration, which is used in clinical practice to adjust the p-calcium concentration, if not analysis of ionised calcium, which measures the unbound calcium concentration, is performed [6]. Analyses of p-calcium concentrations, usually total p-calcium concentrations, are used in primary health care (PHC) to detect primary hyperparathyroidism, cancer and kidney disease [7]. Elevated concentrations are usually correlated to disease [8–10]. However, low concentrations have also been shown to correlate to poor prognosis in hospitalised patients [10,11] as well as in a large population-based study, where an increased cardiovascular mortality in patients with low ionised calcium concentrations was found [12]. We found in an earlier study that both elevated and low p-calcium concentrations were associated with increased mortality [13].

There is increasing evidence in hospitalised patients that low p-albumin concentrations are associated with increased mortality, both in specific disorders such as sepsis or trauma, and in all-cause mortality [14–16]. Furthermore, several population surveys have found increased mortality in patients with low p-albumin concentrations [17–19]. Low p-albumin concentrations seem to be associated with increased cancer, cardiovascular, and all-cause mortality rates in middle-aged males [20]. In one study from the PHC with short follow-up, patients with unexplained low p-albumin concentrations had a slightly increased risk of non-skin cancer during the following 12 months [21].

It is not known if low p-albumin concentrations in patients attending PHC are associated with increased mortality. Furthermore, there are few studies addressing low calcium concentrations and mortality. The aim of this study was therefore to investigate the association between p-albumin and p-calcium concentrations and total mortality in a large PHC setting.

## Material and methods

### Data sources

The patient data were extracted from the computerised patient records used in routine care from all 24 publicly run PHC centres in Skaraborg. Skaraborg is a mostly rural area in the southwest of Sweden, with about 250,000 inhabitants. Between 2001 and 2015, these PHC centres used the same computerised patient record system, ProfDoc Journal III (PDIII, Profdoc AB, Uppsala).

### Study participants

Data were extracted for all patients who had a p-calcium analysis between 1st January 2001 to 31st December 2005 (*n* = 52,099). Data including sex, age, personal identification number, p-albumin and p-calcium analysis (including date of analysis) were extracted using a special built software. In this group, all patients with simultaneous p-albumin and p-calcium analyses (*n* = 44,258) were selected and the results of the first analysis were extracted. Patients with presumably erroneous p-albumin concentrations (below 10 or above 52 g/L) [15] were excluded leaving 44,189 patients. We limited the study to adult patients, 18 years and older leaving 43,052 patients in the analysis. No p-calcium concentrations were excluded. Most p-albumin and p-calcium analyses were analysed centrally in two PHC laboratories by Integra 400 + (Roche Diagnostics Scandinavia AB, Bromma). Until 2006, one PHC centre analysed p-albumin and p-calcium using Vision (Abbott, Solna), and until 2002, one centre used Hitachi 902 (Roche Diagnostics Scandinavia AB, Bromma). All the PHC centre laboratories in the area were certified by the Swedish Board for Accreditation and Conformity Assessment (SWEDAC), assuring adherence to the ISO 15 189 standard, in September 1998.

Using personal identification numbers, we linked the data from the computerised patient records to the National Swedish Cause of Death Register, including the date of death and underlying cause of death (coded according to the International Classification of Diseases, 10th revision, ICD–10, WHO 2016), between 1st January 2001 to 31st December 2005. The follow-up time was calculated as the time from the date of the first p-albumin and p-calcium analyses until the date of death or end of follow-up, whichever came first.

### Statistics

Descriptive statistics for women and men were performed separately. The mean p-albumin concentrations for each year of age were calculated for both sexes. The p-albumin analyses were categorised into three groups, low (10 − 34), normal (35−44), and elevated p-albumin (45 − 52 g/L) [15,18]. As the p-albumin concentration varies with age and between the sexes [5], the included patients were grouped into age groups by quartiles using the age at the first p-albumin analysis (18–47, 48–62, 63–75, and 76–103 years). Kaplan-Meier survival plots were used to compare survival in the three albumin groups stratified by sex and age quartiles. Then, using Cox regression models, hazard ratios (HR) were calculated, with a 95% confidence interval (CI) for the association between total mortality and p-albumin concentration. Separate analyses were performed for each age quartile and sex. Age was included in the separate analyses of the age quartiles as a covariate. The same models were used to calculate the association between cause-specific mortality and the p-albumin concentration for malignant neoplasms (chapter C, ICD–10) and cardiovascular disease (chapter I, ICD–10). Albumin adjusted -calcium concentrations were not used. P-calcium concentrations were also categorised into three groups, low (≤2.30), normal (2.31–2.49) and elevated (≥2.50 mmol/L). Using Cox regression models, HR for the associations between total mortality and p-calcium concentrations were calculated. Two models were used, one unadjusted model and a second model with p-albumin and age included as covariates, but without division into age quartiles, as calcium does not vary with age. All statistical analyses were performed using the IBM SPSS 22.0 statistical package for Windows (IBM Corp., Armonk, NY).

## Results

**Figure 1.**
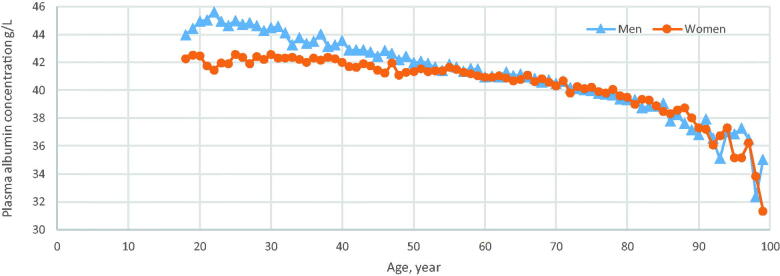
Patients with a plasma albumin analysis in 24 primary health care centres in Sweden in 2001–2005. Mean plasma albumin concentrations for each year of age by sex.

The mean age of the 43,052 patients was 60.7 ± 18.4 years, 16,852 (39.1%) of whom were men, Table 1. More women (60.9%) than men had p-albumin and p-calcium analyses, Table 1. The mean follow-up time was 10.3 ± 4.0 years. The p-albumin concentration decreased with age, in men from the age of 20, in contrast to women where the concentration was stable until 40 years of age and then declined, Figure 1. A low p-albumin concentration was found in 5.4% of all individuals. The mean p-calcium concentration was 2.41 ± 0.11 mmol/L (women, 2.42 ± 0.11; men, 2.40 ± 0.10 mmol/L).

**Table 1.** Patients with plasma albumin and calcium analysis in primary health care between 2001 and 2005, women and men.

	Women					Men				
Age quartile	18–47	48–62	63–75	76 –	Total	18–47	48–62	63–75	76 –	Total
*Total*										
Individuals *n*	6,948	6,332	6,032	6,888	26,200	3,710	4,488	4,496	4,158	16,852
Age^a^, mean ± SD	34.9 ± 8.4	55.4 ± 4.2	69.3 ± 3.8	82.7 ± 4.9	60.3 ± 19.0	35.4 ± 8.4	55.6 ± 4.2	69.1 ± 3.8	82.0 ± 4.5	61.3 ± 17.5
Albumin, mean ± SD	41.8 ± 3.3	41.0 ± 3.1	40.0 ± 3.3	38.4 ± 3.7	40.3 ± 3.6	43.4 ± 3.2	41.3 ± 3.3	40.1 ± 3.2	38.4 ± 3.9	40.8 ± 3.8
Calcium, mean ± SD	2.39 ± 0.10	2.42 ± 0.10	2.43 ± 0.12	2.43 ± 0.12	2.42 ± 0.11	2.43 ± 0.09	2.40 ± 0.10	2.40 ± 0.10	2.39 ± 0.11	2.40 ± 0.10
*Low albumin*^b^										
Individuals *n*	113	134	270	914	1,431	30	114	194	573	911
Age^a^, mean ± SD	33.5 ± 8.1	56.6 ± 3.9	69.8 ± 3.7	85.3 ± 5.5	75.6 ± 16.4	37.5 ± 9.0	56.9 ± 4.0	70.3 ± 3.7	83.6 ± 5.0	75.9 ± 12.7
Albumin, mean ± SD	32.5 ± 2.5	32.2 ± 2.3	31.9 ± 3.1	31.6 ± 2.8	31.8 ± 2.8	30.9 ± 3.9	31.2 ± 3.6	32.0 ± 2.4	31.4 ± 2.9	31.5 ± 3.0
Calcium, mean ± SD	2.30 ± 0.13	2.33 ± 0.11	2.34 ± 0.14	2.35 ± 0.15	2.34 ± 0.15	2.28 ± 0.12	2.31 ± 0.22	2.34 ± 0.16	2.31 ± 0.12	2.32 ± 0.14
*Normal albumin*^c^										
Individuals *n*	5,543	5,470	5,383	5,744	22,140	2,380	3,767	3,991	3,439	13,577
Age^a^, mean ± SD	35.2 ± 8.4	55.4 ± 4.2	69.3 ± 3.8	82.3 ± 4.7	60.7 ± 18.4	36.9 ± 7.9	55.7 ± 4.2	69.1 ± 3.7	81.7 ± 4.4	63.0 ± 16.1
Albumin, mean ± SD	40.9 ± 2.2	40.5 ± 2.3	40.0 ± 2.3	39.2 ± 2.3	40.1 ± 2.4	41.8 ± 1.9	40.8 ± 2.2	40.0 ± 2.3	39.3 ± 2.4	40.4 ± 2.4
Calcium, mean ± SD	2.38 ± 0.09	2.41 ± 0.10	2.43 ± 0.11	2.43 ± 0.11	2.42 ± 0.11	2.42 ± 0.09	2.40 ± 0.09	2.40 ± 0.10	2.40 ± 0.10	2.40 ± 0.10
*Elevated albumin*^d^										
Individuals *n*	1,292	728	379	230	2,629	1,300	607	311	146	2,364
Age^a^, mean ± SD	33.4 ± 8.4	55.2 ± 4.1	68.5 ± 3.6	81.5 ± 4.3	48.7 ± 17.9	32.7 ± 8.6	54.8 ± 4.3	68.3 ± 3.8	80.6 ± 4.2	46.0 ± 17.5
Albumin, mean ± SD	46.4 ± 1.6	46.3 ± 1.5	46.3 ± 1.6	45.9 ± 1.3	46.3 ± 1.5	46.7 ± 1.7	46.3 ± 1.4	46.3 ± 1.5	46.1 ± 1.4	46.5 ± 1.6
Calcium, mean ± SD	2.44 ± 0.10	2.47 ± 0.10	2.49 ± 0.10	2.50 ± 0.10	2.46 ± 0.10	2.46 ± 0.09	2.45 ± 0.10	2.44 ± 0.09	2.47 ± 0.10	2.46 ± 0.10

*n*: number. Reference range albumin 18–40 years 36–48 g/L, 41–70 years 36–45 g/L, >70 years 34–45 g/L. Reference range calcium 2.15–2.50 mmol/L.

^a^Age at sampling time 2001–2005. ^b^Patients with low albumin concentration, 10–34 g/L. ^c^Patients with normal albumin concentration, 35–44 g/L. ^d^Patients with elevated albumin concentration, 45–52 g/L.

### Mortality

**Figure 2.**
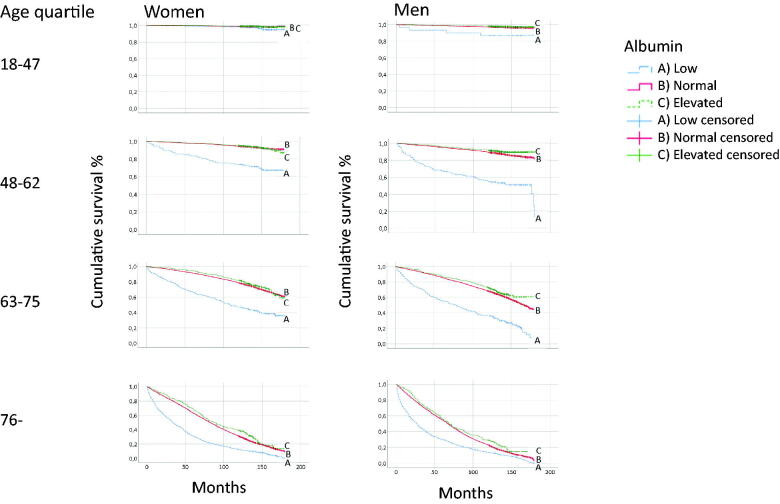
Mortality of patients with low (10–34), normal (35–44) and elevated (45–52 g/L) p-albumin concentrations in 24 primary health care centres in Sweden in 2001–2005.

**Figure 3.**
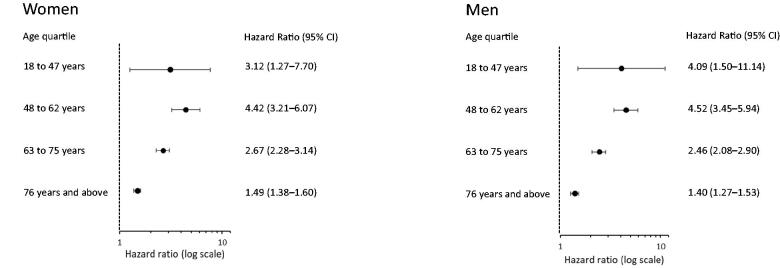
Hazard ratios for mortality with a 95% confidence interval in age quartiles, comparing patients with low (10–34 g/L) and normal (35–44 g/L) plasma albumin. Data for women and men in 24 primary health care centres in Sweden in 2001–2005.

The initial analyses showed that the p-albumin concentration was the most important marker for the prognosis. The p-albumin concentration was therefore analysed separately. Men had a higher proportion of deaths than women, Table 2. Patients of both sexes with low p-albumin concentrations had the highest mortality in all age quartiles compared with patients with normal or elevated p-albumin concentrations, Figure 2. In both women and men, the HR for mortality in the age quartiles was increased at low compared with normal p-albumin concentrations; greatest in the younger quartiles and declining with age, Figure 3. In the group 48–62 years, the HR for mortality was 4.42 (CI 95% 3.21–6.07) and 4.52 (CI 95% 3.45–5.94) for women and men, respectively, compared with 1.49 (95% CI 1.38–1.60) and 1.40 (95% CI 1.27–1.53) in the oldest age quartile.

**Table 2.** Mean follow-up time in patients with plasma albumin and calcium analysis in primary health care between 2001 and 2005 linked to data from the National Swedish Cause of Death Register updated on 31 December 2015, women and men.

	Women					Men				
Age quartile	18–47	48–62	63–75	76 –	Total	18–47	48–62	63–75	76 –	Total
*Total*										
Deaths *n* (%)	113 (1.6%)	516 (8.1%)	1,984 (32.9%)	5,837 (84.7%)	8,450 (32.3%)	123 (3.3%)	669 (14.9%)	2,050 (45.6%)	3,721 (89.5%)	6,563 (38.9%)
Mean follow-up time	149.6	147.0	132.3	80.5	126.8	147.1	140.6	120.9	69.8	119.3
*Low albumin*^a^										
Deaths *n* (%)	5 (4.4%)	42 (31.3%)	165 (61.1%)	888 (97.2%)	1,100 (76.9%)	4 (13.3%)	58 (50.9%)	150 (77.3%)	561 (97.9%)	773 (84.9%)
Mean follow-up time	150.5	126.0	98.2	49.9	74.1	134.8	102.8	81.3	47.6	64.5
*Normal Albumin*^b^										
Deaths *n* (%)	90 (1.6%)	424 (7.8%)	1,720 (32.0%)	4,776 (83.1%)	7,010 (31.7%)	86 (3.6%)	551 (14.6%)	1,792 (44.9%)	3,044 (88.5%)	5,473 (40.3%)
Mean follow-up time	151.3	149.0	134.2	85.0	129.3	150.0	142.7	122.9	73.3	120.5
*Elevated Albumin*^c^										
Deaths *n* (%)	18 (1.4%)	50 (6.9%)	99 (26.1%)	173 (75.2%)	340 (12.9%)	33 (2.5%)	60 (9.9%)	108 (34.7%)	116 (79.5%)	317 (13.4%)
Mean follow-up time	142.3	139.3	129.5	89.3	135.0	142.6	134.8	119.4	76.2	133.4
*Low calcium*^d^										
Individuals *n*	1,199	765	633	942	3,539	311	638	681	852	2,482
Deaths *n* (%)	25 (2.1%)	82 (10.7%)	244 (38.5%)	844 (89.6%)	1,195 (33.8%)	16 (5.1%)	123 (19.3%)	305 (44.8%)	774 (90.8%)	1,218 (49.1%)
Mean follow-up time	148.6	146.7	124.6	69.3	122.8	141.8	133.5	118.6	65.0	107.0
*Normal calcium*^e^										
Individuals *n*	4,770	4,187	3,944	4,266	17,167	2,486	3,127	3,166	2,703	11,482
Deaths *n* (%)	70 (1.5%)	304 (7.3%)	1246 (31.6%)	3571 (83.7%)	5191 (30.2%)	75 (3.0%)	422 (13.5%)	1419 (44.8%)	2399 (88.8%)	4315 (37.6%)
Mean follow-up time	150.1	147.7	134.1	82.6	129.1	147.8	142.6	122.3	71.4	121.4
*Elevated calcium*^f^										
Individuals *n*	979	1,380	1,455	1,680	5,494	913	723	649	604	2,889
Deaths *n* (%)	18 (1.8%)	130 (9.4%)	494 (34.0%)	1422 (84.6%)	2064 (37.6%)	32 (3.5%)	124 (17.2%)	326 (50.2%)	549 (90.9%)	1031 (35.7%)
Mean follow-up time	148.4	145.2	130.5	81.5	122.4	147.2	138.4	116.2	69.4	121.8

Percentage of deaths within the age groups.

^a^Patients with low albumin concentration, 10–34 g/L. ^b^Patients with normal albumin concentration, 35–44 g/L. ^c^Patients with elevated albumin concentration, 45–52 g/L. ^d^Patients with low calcium concentration, ≤ 2.30 mmol/L. ^e^Patients with normal calcium concentration, 2.31–2.49 mmol/L. ^f^Patients with elevated calcium concentration, ≥ 2.50 mmol.

An increased HR for mortality in both cardiovascular and malignant diseases was shown in women and men with low compared with normal p-albumin concentrations. Mortality was greatest in the younger quartiles. In women, the HR was 7.56 (95% CI 1.73–33.0) in the ages 18–47 years, and 1.30 (95% CI 1.17–1.44) in the oldest age quartile for cardiovascular diseases. In malignancies, the HR was 2.94 (95% CI 1.68–5.16) in the ages 48–62 years, and 1.59 (95% CI 1.25–2.20) in the oldest age quartile. Correspondingly, in men, the HR was 3.46 (95% CI 2.04–5.87) in the ages 48–62 years, and 1.30 (95% CI 1.14–1.48) in the oldest age quartile for cardiovascular diseases, and in malignancies, the HR was 3.80 (95% CI 2.34–6.15) in the age quartile 48–62 years, and 1.83 (95% CI 1.48–2.28) in the oldest age quartile.

In the unadjusted Cox regression analysis, low p-calcium concentrations were associated with an increased HR of death compared with normal p-calcium concentrations, 1.31 (95% CI 1.25–1.37), which disappeared when adjusting for p-albumin, age and sex, 1.01 (95% CI 0.96–1.05). Data not shown. However, in patients with elevated p-calcium concentrations, the increased HR of mortality, 1.15 (95% CI 1.10–1.20), was almost unchanged after adjusting for p-albumin, sex and age 1.17 (95% CI 1.13–1.22).

## Discussion

### Principal findings

We found that a low p-albumin concentration at baseline was a risk marker for total mortality, as well as mortality in malignancies and cardiovascular disease after 10–14 years, especially at younger ages. Elevated but not low p-calcium concentrations were also associated with increased mortality.

### Strength and weaknesses of the study

The strength of this study was the long follow-up and the large number of subjects. Moreover, the data from the Swedish National Cause of Death Register are comprehensive, comprising almost all the inhabitants (99%) that have died and their cause of death [22]. A study showed 77% agreement between the registered cause of death and the results from a medical record investigation, with the agreement being higher in the younger age quartiles for malignant neoplasms [23].

One weakness was that the study was originally designed to study the risk of low p-calcium concentrations, not the risk of low p-albumin concentrations. However, when the p-albumin concentration was included in the analyses, the association between p-calcium concentrations and increased mortality disappeared, and only elevated p-calcium concentrations remained significant. P-albumin appeared to be the major marker driving the high mortality risk in patients with low unadjusted p-calcium concentrations. Consequently, we analysed the association between p-albumin concentrations and mortality. A post-hoc analysis showed that 90% of all p-albumin analyses were accompanied by p-calcium analyses. A single analysis of p-albumin was often included in protein electrophoresis and investigations of liver pathologies. In our opinion it was reasonable to study the association between the p-albumin concentration and mortality, as our large cohort included almost all patients (90%) with a p-albumin analysis.

Another weakness was that we compared patients with abnormal p-albumin and p-calcium concentrations with patients with normal concentrations attending primary health care, presumably for symptoms occasioning the analysis. A comparison with a sample from the background population would have been preferable. Data on morbidity, pharmacological treatment or the reasons for analysing p-albumin and p-calcium were not included in the study. However, we assume that many of the patients with normal calcium and albumin concentrations are reasonably healthy, as increased use of these analyses have been proposed for a long time [24,25], and around 20% of the background population had a calcium analysis during the study period.

The youngest age quartile with low p-albumin was small, the confidence intervals were wide, and the results should thus be interpreted with caution. Finally, as the study was observational, we can only study associations between risk markers and outcome and not causality.

### Findings in relation to other studies

An increased cardiovascular disease mortality in patients with low ionised serum calcium concentrations was found in an American population survey of calcium and vitamin D supplementation and prognosis [26]. Our study was similar in size, but we could not replicate the finding of increased mortality in patients with low p-calcium concentrations. One possible explanation may be the different methods employed to recruit the studied populations. Our study consisted of patients whose median age was 15 years higher and our population included a greater proportion of women. Presumably, the most important difference between our and the American study was the use of total p-calcium in our study instead of ionised calcium. Total p-calcium analysis was the standard procedure in primary health care during the study period [24]. The concentration of calcium in the blood is better reflected by ionised calcium than by p-albumin-adjusted total p-calcium [27]. Therefore, ionised calcium is now recommended, especially in the follow-up of aberrant results of total p-calcium analyses [27].

P-albumin concentrations decreased with age, earlier in men than in women, even though women had lower p-albumin concentrations than men from 20 years of age until the menopause. This is in line with a study from PHC in the United Kingdom [5]. This difference may have hormonal causes; a hypothesis that is strengthened by findings of a decrease in p-albumin by 15–20% during the second and third trimesters of pregnancies [28]. In this study, 5.4% of the included individuals had low p-albumin concentrations (< 35 g/L), while, with the same p-albumin definition, a lower prevalence was found in a Japanese cross-sectional study of women and men above 65 years of age; 1.5% and 2.4%, respectively [18]. This difference may be explained by the fact that the Japanese study was population-based whereas our population included individuals attending the health service for a condition that warranted an analysis.

In our study, low p-albumin concentrations in both sexes were associated with the highest mortality, which is in line with an earlier population study [17], in which the individuals were older than 71 years. Both total and cause-specific mortality were greatest in the younger quartiles in both women and men with low compared with normal p-albumin concentrations. The increased mortality could be due to complex associations with underlying morbidity and frailty [29]. However, p-albumin is not an independent risk marker by itself but may reflect acute or chronic inflammation as found in a recent study of risk of cardiovascular disease [30]. Different responses from a variety of diseases activate acute-phase protein reaction, which may cause decreased synthesis, malabsorption, abnormal loss [3], or transcapillary escape of albumin [1,2], leading to low p-albumin concentrations [1,2], which might reflect the underlying causes.

### Meaning of the study

The p-albumin concentration could be used as a clinical marker to identify patients with increased risk of a poor long-term prognosis, especially in middle-aged individuals. Physicians in PHC should therefore pay attention to patients with low p-albumin concentrations and promptly take diagnostic measures in order to find possible underlying causes.
